# Remote labs in higher engineering education: engaging students with active learning pedagogy

**DOI:** 10.1007/s12528-022-09331-4

**Published:** 2022-08-12

**Authors:** Antoine Van den Beemt, Suzanne Groothuijsen, Leyla Ozkan, Will Hendrix

**Affiliations:** Eindhoven University of Technology, Eindhoven, The Netherlands

**Keywords:** Engineering education, Active learning, Engagement, Case study, Remote lab.

## Abstract

In engineering education laboratories serve as experiential learning aimed at engaging students. The past decades saw an increased use of online laboratories, including virtual and remote labs. Remote labs, providing online interfaces to physical labs, allow students to conduct experiments with real-world equipment anywhere and at any time. However, this advantage challenges active student engagement. Little evidence is available on effective pedagogies for student engagement in remote labs. This paper aims to identify how a remote lab assignment based on active learning pedagogy in higher engineering education supports student engagement, with the overarching aim to promote students’ transfer skills from theory to practice. Our research question, “What impact does an active learning pedagogy have on students’ engagement with a remote lab?“, was answered with a case study of two courses on systems and control in higher engineering education. Data included digital traces, course evaluations, interviews, and observations. Students reported how remote labs, to be used anywhere at any time, require self-regulation and scheduling of experiments. However, accompanying open-ended lab assignments encouraged students to engage with the lab and the theoretical content of the course by creating a ‘need-to-know.’ Our results furthermore suggest the need for a structured arrangement of open-ended lab assignment, lab preparation, teamwork supporting peer learning and discussion, progress meetings focused on feedback and formative assessment, and reports focused on reflection. Engagement can be strengthened by support for students before and during the experiments, clear signposting about the experiment and lab set-up, and pre-structuring of lab activities.

## Introduction

In response to today’s social, economic, and environmental complex challenges, higher education increasingly embraces context-rich approaches to prepare engineering students for future challenges (Chen et al., 2021; Kohn Rådberg et al., 2020). Such context-rich approaches might include experiential learning (Abdulwahed & Nagy, 2009; Li et al., 2019), which is reported to increase academic achievement and the transfer from theory to practice (Chen et al., 2021). To promote these transfer skills, contemporary theories advocate explicit connections between abstract ideas and concrete representations (Fyfe & Nathan, 2019).

In engineering education, laboratories are used often as a form of experiential learning aimed at engaging students (Bernhard, 2010; Krivickas & Krivickas 2007). Student engagement, is shaped amongst others by the interplay between the learning environment, including laboratories, learning activities, and relationships (Bond et al., 2020). Engagement can be observed via cognitive, behavioural, or affective indicators. The argument is that in an iterative process active student engagement accelerates transfer from theory to practice, leading to short- and long-term outcomes, which in turn fuel engagement.

Traditionally, engineering education applies physical laboratories to promote transfer skills among students (Feisel & Rosa, 2005). In physical labs, students engage with real equipment, gain hands-on skills and are confronted with the non-ideal characteristics of real-world systems. The past decades saw an increased use of online laboratories, specifically virtual and remote labs (Grodotzki, Ortelt, Tekkaya, 2018). Virtual labs deliver all content and the experiment in a virtual environment, with the help of videos and simulations (Bhute et al., 2021). In virtual labs, students can run simulations of experiments from any location at any time. Non-ideal characteristics of real-world experiments are often absent in virtual laboratories (Lowe et al., 2008), although virtual labs can support experimentation with non-observable phenomena by making them visible (Heradio et al., 2016). Virtual labs offer 24/7 availability to students, giving them more opportunities to conduct experiments than physical labs. Furthermore, virtual labs are argued to support active student learning because they elicit iteration of experiments (Heradio et al., 2016; Kirschner, 1988). Remote labs, providing online interfaces to physical labs (Bhute et al., 2021; Zubia & Alves, 2011), seem to offer the best of both worlds. Students can conduct experiments with real-world equipment from anywhere and at any time, offering both the non-ideal characteristics of a physical lab and the time and place independent learning of a virtual lab. The essential set-up of a remote lab includes a live video and audio feed so that students can view experiments and controls in real-time without handling the experiment in-person (Bhute et al., 2021).

Research shows that students’ learning outcomes in remote labs are equal or better compared to physical labs (Brinson, 2015; Corter et al., 2011; Heradio et al., 2016; Post et al., 2019). In their review study on the effects of remote labs on cognitive, behavioural, and affective learning outcomes, Post and colleagues (2019) found a positive correlation between students’ learning outcomes and their engagement with remote labs. For example, it was reported that students working with a remote lab conducted more experiments and achieved higher test scores compared to students in a regular lab (Morgan et al., 2012). Other studies confirmed that students with high test scores were also the ones who conducted the most experiments in the remote lab (Broisin et al., 2017; Viegas et al., 2018). Furthermore, students’ use of a remote lab appears to be independent from their perception or satisfaction with the lab (Viegas et al., 2018). This suggests that increased learning gains can result from increased engagement with a remote lab, even when extrinsically motivated by being mandatory.

Building on these findings, it seems paramount to activate students for engaging with remote labs to increase their conceptual understanding. However, the major advantage of online labs, namely being accessible anyplace and anytime, provides a serious challenge to actively engaging students. In a remote lab, students are expected and required to show self-regulation by planning and executing experiments themselves (Daradoumis, Marques Puig, Arguedas, Calvet Linan, 2021; cf., Litzinger, lattuca, Hadgraft, Newstetter, 2011). Furthermore, conducting remote lab experiments from anyplace, lacks the presence of teachers and peers to encourage, motivate and support students in these experiments. This contrasts with physical labs with designated timeslots to conduct experiments, and teachers or lab assistants available for support.

The differences in characteristics between physical and online labs require reconsideration of existing lab pedagogies, including how to actively engage students and consequently promote intended academic achievement and transfer skills. Existing literature on remote labs typically provides detailed descriptions of the required technology to make physical lab systems remotely accessible (e.g., Sus et al., 2021; Mohammed et al., 2020). And although studies emphasise the need for additional technology to support student engagement and learning (e.g., Bhute et al., 2021), little evidence appears available on effective pedagogies for new forms of labs to promote student engagement and learning. Student-centred approaches, such as active learning, are perceived as engaging students in learning through interactions, including reading, watching, listening, analysing, or experimenting (Kalinga & Tenhunen, 2018; Nascimento et al., 2019; Shehkar, Prince, Finelli, Demonbrun, & Waters, 2019). In the process of active learning, students are involved in knowledge creation rather than knowledge provision (Cattaneo, 2017; Fields et al., 2021).

This paper aims to identify how a remote lab assignment based on active learning in higher engineering education supports student engagement. By studying student engagement with a remote lab in two higher engineering systems and control courses, this study sets out to contribute to detailed and evidence-informed knowledge on online lab pedagogy.

## Active learning in higher engineering education

Higher engineering education institutions increasingly make their educational programmes open, flexible, and context-rich (see also Gallagher & Savage 2020). These context-rich programmes share a design of assignments to engage students via activating, self-directed work scenarios (Johnson et al., 2009). The goal of these assignments is to learn how to address the problem and to learn what it takes to work towards a solution, rather than to solve the problem itself.

These self-directed work scenarios support active learning by urging students to construct a network of knowledge and take ownership of their own learning process (Hernández-de-Menéndez, Vallejo Guevara, Tudón Martínez, Hernández Alcántara, & Morales-Menendez, 2019; Trimingham et al., 2016). Central elements to active learning are (1) physical spaces, (2) technology, including Internet access and lab equipment, and (3) activities, including lab activities and dialogue, together leading to (4) results, such as an enhanced conceptual understanding and improved student performance (Hernández-de-Menéndez et al., 2019).

Active learning pedagogy connects in-class learning with authentic, field- and practice-based experiences (Salisbury & Irby, 2020). It focuses on both process and content, and strives for interdisciplinarity and collaboration (Cattaneo, 2017). Teaching strategies for active learning include group discussions, problem solving, case studies or structured learning groups (Kalinga & Tehunen, 2018). Teachers take on a facilitating role by converting learning into an authentic and meaningful process (Vodovozov et al., 2021). Active learning is built on formative assessment with reflection, feedback, and support, rather than on summative assessment (Cattaneo, 2017). Reported benefits are for instance improved critical thinking skills, increased retention and transfer of new information, increased motivation, and improved interpersonal skills (Winarno, Muthu, & Sing, 2016).

Despite these benefits, active learning has seen a slow adoption in classrooms (Jamieson & Lohmann, 2012), caused by student resistance and lack of engagement (Froyd et al., 2013). Active participation required from students often conflicts with their expectations about learning activities (Shehkar et al., 2019). This conflict can result in resistance, such as offering excuses for not doing assignments, pretending to comply, poor task completion, or voicing concerns or objections (Weimer, 2013). Furthermore, it is reported how students in higher engineering education prefer ‘passive’ tools, such as webinars and asynchronous recording of demonstrations, to active learning activities, such as online direct connections with laboratory set ups (Ozadowicz, 2021; Vodovozov et al., 2021).

Yet, at the same time, research on the active learning approach challenge-based learning (Malmqvist, Kohn Rådberg, & Lundqvist, 2015; Van den Beemt, Van den Watering, & Bots, 2022) shows how assignments that allow for flexibility and openness, and that support the transfer from theory to practice, create high student motivation and engagement (Van den Beemt & MacLeod, 2021). This finding confirms research that shows how active learning is effective in improving students’ attention and can engage students in taking a deep approach to their learning (Biggs & Tang, 2011; Li et al., 2019;). However, it remains unclear how these findings can be enhanced with an active learning pedagogy for remote labs.

In a search for effective pedagogies for remote laboratories in engineering education, Gustavsson et al., (2009) address major points of attention: introduce specific learning objectives for laboratories in courses that include laboratory components, implement individual student assessment, and introduce free access to online experimental resources as a supplement to traditional laboratories. De Jong et al., (2014) advocate an inquiry learning pedagogy for online labs, including orientation, conceptualization, investigation, and conclusion phases, supported by ongoing discussion (see also Litzinger et al., 2011). Additionally, for active learning lab pedagogies, a sequence of learning activities has been proposed by Abduhlwahed and Nagy (2009), consisting of pre-lab sessions, pre-lab test, lab experiment, and post-lab test including reflective components.

## Research question

In line with the aim of our study and the residing call for verification of remote laboratories (Feisel & Rosa, 2005) and related pedagogies (Lopes et al., 2021), we formulated our research question as:

*What impact does an active learning pedagogy have on students’ engagement with a remote lab?*

We address this research question with a case study of a remote lab assignment and accompanying active learning pedagogy, while acknowledging the goal of engagement as promoting transfer skills from theory to practice (Bond et al., 2020; Bernhard, 2010).

## Case description: implementation of a remote lab following active learning pedagogy

The remote lab in this case study, developed at a university of technology in the Netherlands, consisted of three different physical set-ups. These three set-ups were applied in multiple courses about systems and control. These courses were theoretical in nature with extensive coverage of mathematical topics. To promote students’ conceptual understanding and transfer from theory to practice, the remote lab was introduced in these courses as a practical component. The description below of the remote lab and its implementation in education follows the common elements for active learning as identified by Hernández-de-Menédez and colleagues (2019), namely physical space, technology, activities, and results.

### Physical space

**Fig. 1:**
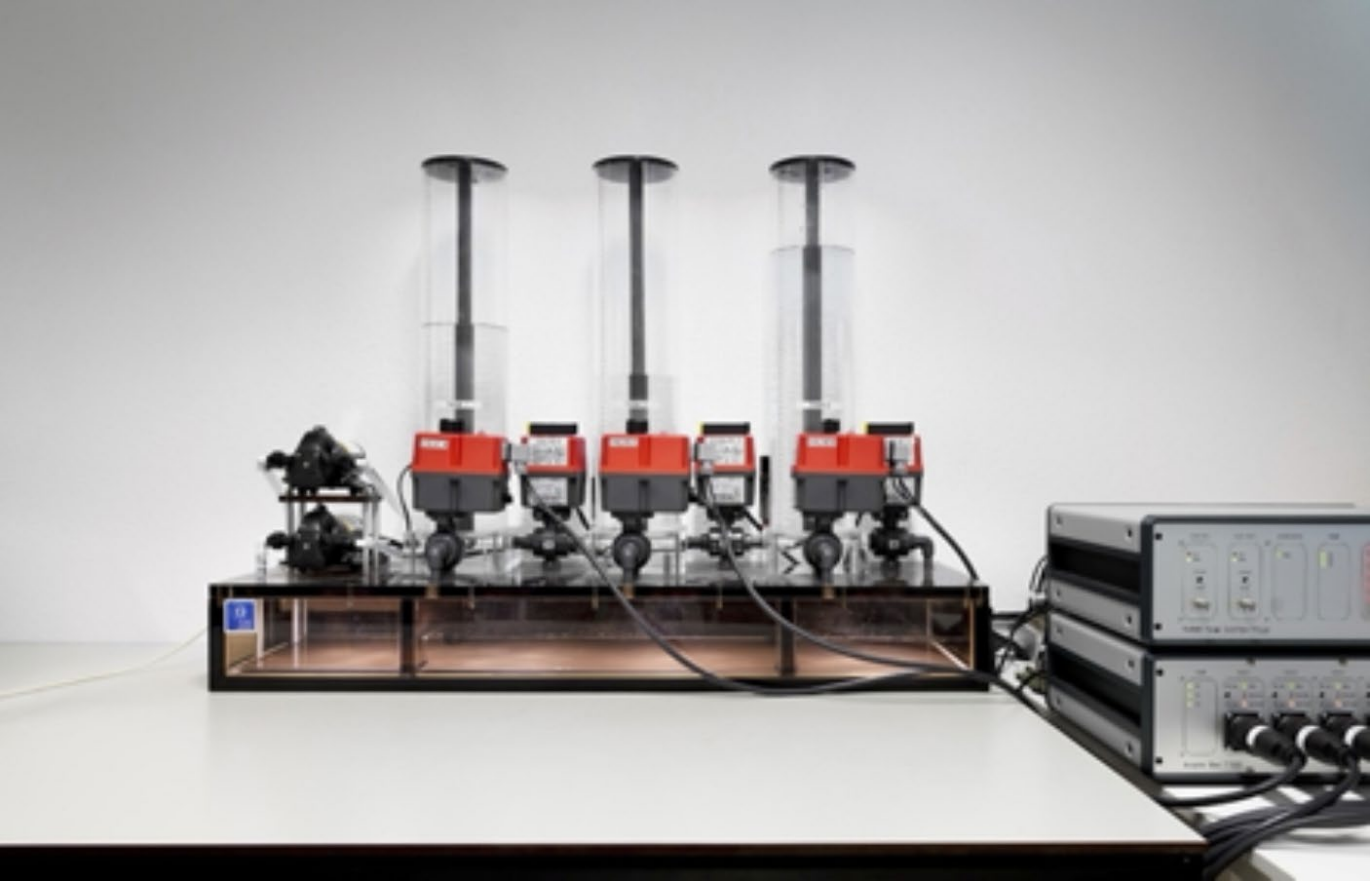
Three-tank set-up

**Fig. 2:**
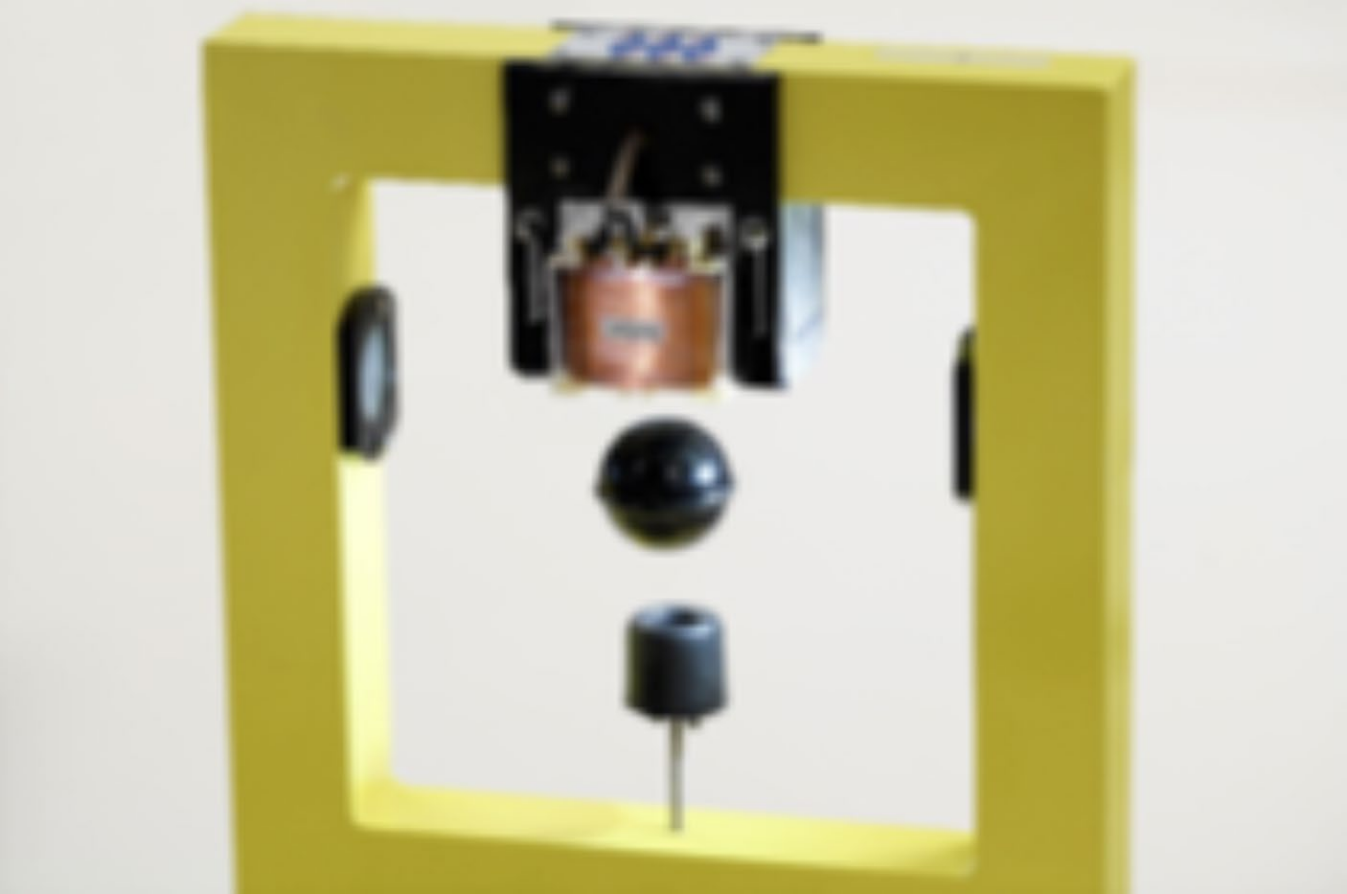
Magnetic ball levitation set-up

**Fig. 3:**
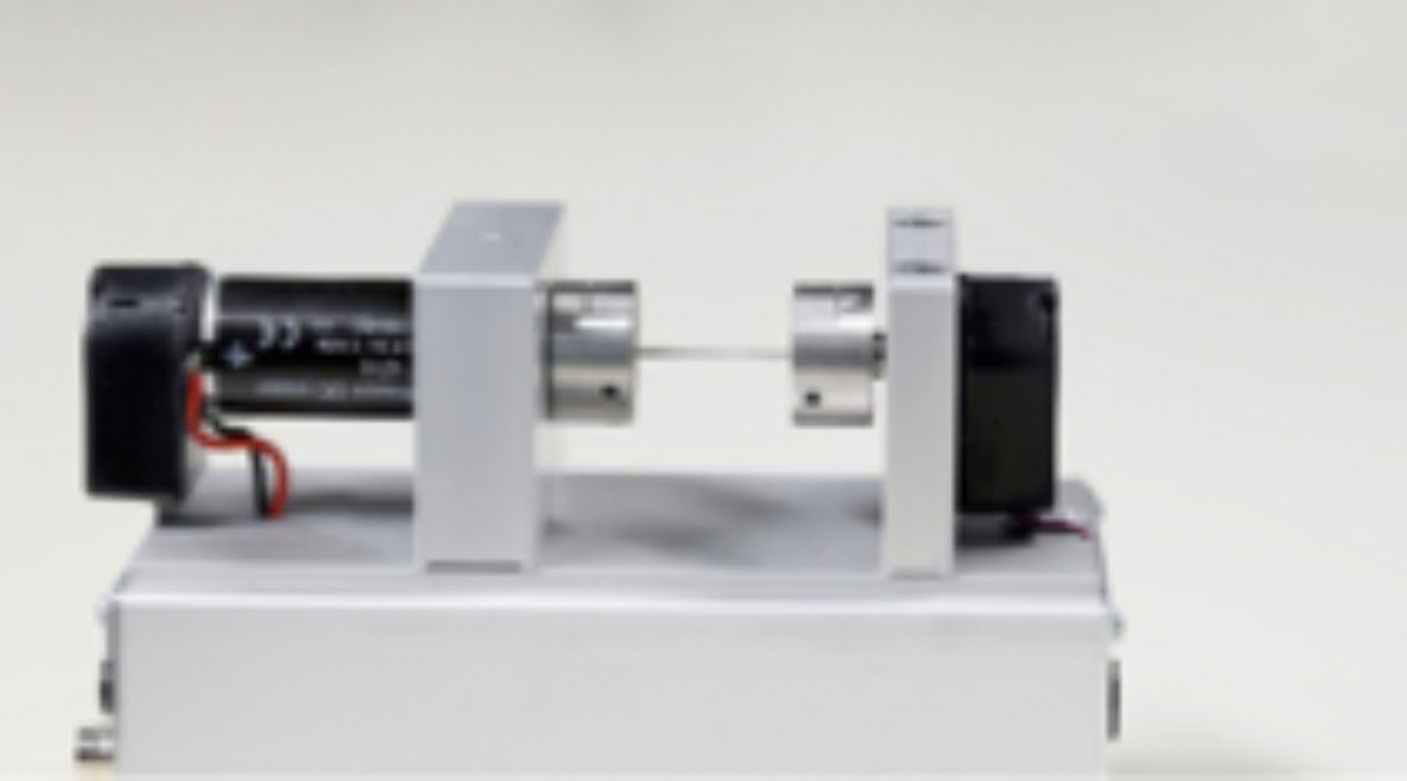
Flexible axle drive set-up

The remote lab consisted of three physical set-ups to be used for systems and control experiments:

1. Three-tank set-up in which water levels had to be maintained within specified limits by operating pumps and valves, while real-time feedback on the water levels was provided (Fig. 1);
2. Magnetic ball levitation set-up in which a metal ball had to be levitated and position controlled in an electromagnetic field (Fig. 2);
3. Flexible axle drive set-up in which the angular positions of two masses were measured with sensors, and students designed and tested speed and position controllers for the masses (Fig. 3).

### Technology

A remote lab includes a physical experiment set-up, a user tracking system, a repository of information for students and staff, and a user-interface (Bhute et al., 2021). The remote lab used in this study was designed to be scalable in number of physical set-ups, students, and courses, and allowed for individual assignments rather than all students conducting the same experiments. The tracking and repository systems consisted of two servers: a master server dealing with login accounts, user rights, administrative and organisational tasks, and a lab server managing the scheduling queues and communication with the physical set-ups. The remote lab could be accessed by students for experimentation through a web-based interface. Video live streams allowed students to observe the physical lab set-up while they conducted experiments.

The user tracking system followed the students’ progress. This progress was recorded as digital traces including date and timestamp, number of experiments, duration of experiments, and success or failure of experiments. The system checked the students’ answers to experiment preparation questions and gave basic advice for the experiment process.

All remote lab set-ups allowed for live interaction experiments and queued automated experiments. For live interaction experiments, data files were uploaded, followed by real time running of the experiment, and downloading and evaluating the results. Live interaction experiments were conducted during a reserved timeslot, which allowed for multiple experiments during that timeslot. Students could reserve timeslots on a 24/7 basis. Queued automated experiments required a timely upload of data files, followed by an automated run of the experiment. Results could be downloaded any time after the experiment had finished.

### Activities

Open-ended lab assignments were designed for students to conduct experiments in the remote lab. The lab assignments were similar for all three physical lab set-ups and consisted of three experiment stages: (1) manually controlling the remote lab experimental set-up, (2) determining constants, and (3) controlling the remote lab experimental set-up. Each of these stages followed the sequence of learning activities as proposed by Abdulwahed & Nagy (2009): pre-lab activities, remote lab experiment, and post-lab activities. Students conducted the lab assignment in groups of two to five students. All learning activities reflected an interplay between the lab environment, learning activities, and relationships to promote student engagement (Bond et al., 2020). Introduction of the remote lab was done at the start of a course via a demonstration and a user manual.

For the first experiment stage, the pre-lab activity consisted of design tasks on modelling and control in a virtual lab (i.e., Matlab Simulink). Students prepared Matlab files and tested them in a virtual representation of the physical set-up before using the files as input for subsequent experiments in the remote lab. The second pre-lab activity was a formative quiz intended to create interaction between teacher and students concerning the theoretical part of the course. The quiz supplied feedback to the teacher on which topics were unclear for students. The following experiment in the remote lab allowed for manually controlling the experimental set-up. This experiment aimed at students experiencing the need for a controller, because manual control cannot provide satisfying results. The experiment was followed by a progress meeting as post-lab activity, with the purpose to either check whether students had started using the remote lab or encourage them to start. Topics from the formative quiz that were found to be unclear for students were also explicitly addressed in the progress meeting to further promote conceptual understanding and transfer from theory to application in the lab assignment.

The pre-lab session for the second experiment stage again consisted of preparing Matlab files for subsequent experiments in the remote lab. These Matlab files could be tested in the virtual lab. However, this was not mandatory, and files could also be directly used in the remote lab. The experiment was aimed at figuring out constants of the physical set-up, needed as input for building a controller in the next experiment stage. Post-lab activities consisted of a progress meeting with the teacher, and students had to draft an intermediate report on the remote lab assignment. For the third experiment stage, aimed at building a controller and experimenting with controlling the experimental set-up, pre-lab activities, remote lab experiments and post-lab activities were similar in nature to the second experiment stage. The lab assignment was concluded with a final report.

The progress meetings served as a form of formative assessment. They were intended to provide feedback on the conducted experiments, achieved results, and preliminary versions of the intermediate and final reports. The progress meetings also served to support continuation of experiments.

The intermediate and final reports served as a form of summative assessment; they were graded and counted toward the students’ final course grade. Both reports followed the steps from preparation of the experiment, to running the experiment, and evaluating and reflecting on the results. The report was also intended for students to learn how to draft a technical report, formulate a line of argument, and reflect on options and choices in experiments. Additionally, the reports had to include seminal literature on the experiment set-up as an exercise to find and review empirical literature.

### Results

The intended outcomes for students focused on developing an understanding of how theoretical, mainly mathematical, topics can be applied in practice. For example, the Bachelor course “Process Dynamics & Control” aimed to introduce chemistry students to the theory and practice of modelling chemical process dynamics for the purpose of control system design. After this course, students should be able to:

- Use knowledge of mathematics to understand and analyse main mechanisms that govern dynamics of chemical processes;
- Analyse and interpret transient and frequency response data;
- Design, validate and tune conventional feedback controllers for chemical processes such as reactors and distillation columns;
- Use tools such as Matlab and Simulink for analysis and design of process models and control systems.

## Method

A case study (Yin, 2009) was conducted on the remote lab and assignments based on an active learning pedagogy as described above. Complementary quantitative and qualitative data from two courses on systems and control in higher engineering education were collected and analysed to identify how the remote lab and assignments supported student engagement. The purpose of this evaluative case study was to determine the worthwhileness of the case, as judged by the researchers, and to convey the findings to interested audiences (cf. Bassey, 1999).

### Context

The case study was conducted at a Dutch university of technology. The remote lab set-ups and assignments were implemented in several engineering courses on systems and control in two departments. In this study, we focus on the implementation of the three-tank remote lab set-up in the course “Process Dynamics & Control” at the chemical engineering department which ran Spring of 2020 and 2021, and the integration project “Systems and Control” at the electrical engineering department, which ran Spring of 2020. An overview of the courses and student numbers is provided in Table 1.

**Table 1:** *Courses and students for the three-tank remote lab set-up*

Course		Students
Integration project “Systems and Control”, Electrical Engineering	Spring 2020	58 students, groups of 4 students
Course “Process Dynamics and Control”, Chemical Engineering	Spring 2020	57 students, groups of 5 students
Course “Process Dynamics and Control”, Chemical Engineering	Spring 2021	48 students, groups of 5 students

## Data collection

Data were collected from multiple perspectives and multiple sources providing source and method triangulation with the aim to enhance the internal validity of results (Miles & Huberman, 1994; Stake, 2005). Data included digital traces by students in the remote lab user tracking system, results of students’ course evaluations, interviews with the teacher and with four students, course materials, and notes of the observations of progress meetings.

*Digital traces* were collected to identify students’ activities in the remote lab, mainly concerning number and timing of conducted live and automated experiments. The *results of students’ course evaluations* and the interviews with the students provided insight into students’ learning experiences and use of the remote lab. The course evaluations were conducted after conclusion of the courses. They were filled out by 36 of 58 students from the integration project “Systems and Control”, by 20 of 57 and 17 of 48 students from the course “Process Dynamics and Control” in 2020 and 2021 respectively. In the course evaluations students were asked about the perceived added value of the remote lab for the course, preferences for remote or physical labs, and user friendliness and availability of the remote lab. The results were summarized by the first author and checked by the second author. The summary was found to be accurate.

The *interviews with four students* were conducted at the end of the “Process Dynamics and Control” course Spring 2021. The students volunteered to take part in the interviews upon a request by the second author. During the interviews, the students were asked to reflect on their learning experience and process of using the remote lab. The *interview with the teacher* of the “Process Dynamics and Control” course focused on considerations of pedagogy and course organisation. The second author conducted all interviews. The interviews were recorded and summarized individually by the first and second author. The summaries were discussed between the authors. The summaries aligned and no contradictory results were found. The *course materials* and *notes of the observations of progress meetings* were used to further verify and extend the findings.

## Data analysis

In line with the aim of the study, data were analysed to identify how the remote lab assignment contributed to student engagement. Quantitative analysis of students’ digital traces in the remote lab user tracking system was conducted to identify the number of successful experiments divided over live and automated experiments, and time of day. Qualitative content analysis was conducted on the summaries of the course evaluations, the student and teacher interviews, and the course materials and notes of the observations of progress meetings. Specific points of attention in the analysis of all qualitative data were how pedagogy supported engagement, and contextual and flexible learning processes. Data analysis was guided by the elements of engagement, including learning environment, learning activities, and relationships (Bond et al., 2020), and the elements of active learning in engineering education, including physical space, technology, activities, and results (Hernández-de-Menédez et al., 2019).

This study followed the research guidelines of social scientific studies from Eindhoven University of Technology (2014), and the Association of Universities in the Netherlands (2014). Participants took part voluntarily and gave informed consent.

## Findings

This study addressed what impact an active learning pedagogy has on students’ engagement with a remote lab, with the extended purpose to promote students’ transfer from theory to practice. The description of the results is structured following the elements proposed by Bond and colleagues (2020) to promote student engagement: learning environment, learning activities, and relationships.

### Learning environment

For the Integration project, 58 students worked in teams of four. The three tank lab set-up saw 1944 successful experiments, of which 1184 were live, and 760 were queued experiments (Table 2). An experiment could only successfully run if students uploaded files that were compatible with the remote lab and included all information in the experiment definition for the remote lab to build and execute the experiment. During the course “Process Dynamics and Control” in 2020, 48 students in teams of five, have conducted 764 successful experiments, consisting of 199 queued and 565 live experiments. In 2021, 54 students working in teams of five, conducted 437 successful experiments of which 238 were live, and 199 were queued. In all courses the majority of experiments were conducted live, although the numbers of queued experiments show a need for flexibility in scheduling.

**Table 2:** Live and automated experiments and reserved timeslots for three courses

	Integration project “Systems and Control” (2020)	Course “Process Dynamics and Control” (2020)	Course “Process Dynamics and Control” (2021)
*Experiments*			
Successful Queued	760	99	199
Successful Live	1184	565	238
*Timeslots*			
Morning	225	126	58
Afternoon	241	174	83
Evening	170	88	10
Night	78	19	4

Live interaction experiments booking of timeslots showed that the afternoon was most popular for all courses. However, if we combine the evening and night, the availability of the experiment outside office hours appears to fulfil the need for flexibility in scheduling, at least for the integration project. This was confirmed by student evaluations for this course, for example:

“With the flexibility I might be able to work at a time when I suddenly have some new ideas.” *(Student course evaluation integration project “Systems and Control”)*

Yet, students also reported preference for more flexibility in experiment duration beyond the fixed time currently applied. Also, the opportunity to perform multiple cycles of experimentation was often used. Students reported a preference for the remote lab over the physical lab. However, evaluations also showed the need for scaffolding and support for the process and the software use.

Student evaluations of the integration project showed that around two-third of the students agreed that the remote lab had added value over simulation of an experiment. A similar percentage of students reported to be in favour of flexibility in scheduling, especially for automated runs because of time efficiency and the possibility to run multiple experiments:

“It is very time efficient to upload an experiment and come back after a day and see that the experiments have run. This takes away the waiting time and makes it really easy to schedule time for working on the project.” *(Student course evaluation integration project “Systems and Control”)*

Some students reported to prefer a live set up, because of the engagement of being present in the lab. The majority of students (72%) reported a positive evaluation of user friendliness of the remote lab, while over 50% reported the user interface to be intuitive.

Students reported how the particular characteristic of a remote lab to be used 24/7 from any location affected their engagement. Since all timeslots were unsupervised by a teacher or lab assistant, students had to show self-regulation and schedule their own experiments. Furthermore, the assignment seemingly allowed endless opportunities to conduct experiments. The students from the Process Dynamics & Control course reported that, following this liberty, they tended to procrastinate because there was always ample opportunity left to conduct experiments.

“I noticed that because of the possibility to plan your own experiments, I tended to procrastinate of just refrain from doing experiments. I didn’t feel any pressure to start with experiments early on, because there was still so much time and opportunity to conduct experiments later on.” *(Student interview “System Dynamics and Control” course 2021)*

The physical set-up of the remote lab included a video camera. Video livestreams from this camera showed students what happened during experiments. This promoted engagement, because students perceived the livestream as an interaction with the remote lab, and it contributed to their understanding of the physical set-up, the experiments, and the results. Students from the Process Dynamics & Control course reported to perceive this as very valuable, as illustrated by this quotation from one of the student interviews:

“It is nice that there is a camera, because you can see that the valve is moving and how fast that goes. You can also see that there is some response time before the valve opens up, that it doesn’t happen instantaneously. The video feed shows that well. I think that is valuable. So even when you are not sitting next to the lab set-up, you can still see exactly what happens.” *(Student interview “System Dynamics and Control” course 2021)*

### Learning activities

The learning activities started from an open-ended lab assignment. Students from the Process Dynamics & Control course reported that this assignment encouraged them to engage with the remote lab and the theoretical content of the course by creating a ‘need-to-know.’ By conducting experiments, students encountered unknown issues required to continue with the next step of their lab assignment. For example, one issue concerned the need for a controller for improved control of the experimental set-up when manual control does not give satisfactory results. Another issue covered the need to identify the values of two constants in the three-tank set-up to actually design a controller.

“We worked on developing our own model of the three-tank set-up. When we did that, we noticed that we were missing two constants. So, then we tried to figure out how we could identify those constants.” *(Student interview “System Dynamics and Control” course 2021)*

Learning activities reported by the students Process Dynamics & Control to encourage engagement with the remote lab are the progress meetings and the reports. During the progress meetings, students reported receiving feedback on their work and discuss with the teacher how to continue experimentation. Students considered these meetings to be valuable, because they provided insight in the students’ progress and what subsequent experimental steps could be. According to the students, the progress meetings also forced a deadline to conduct experiments so they actually would have results to discuss with the teacher. The teacher confirmed how the progress meetings were explicitly designed for these purposes: to inform students if they were on the right track, and to prevent procrastination and consequent accumulation of workload towards to end of the course. Preventing accumulation of workload was also important to protect the physical experimental set-up, which is unable to cope with a large number of experiments over a limited period. Prevention of procrastination is additionally intended by the teacher to increase the overall number of experiments students (can) conduct.

“The progress meetings provide a structure for students, because they have to do something and show that in the progress meetings. It encourages students to think about the project. Without the progress meetings, students will leave the project to the last minute. […] The progress meetings help to spread to workload over the duration of the course, which is beneficial for the students and the remote lab system. The progress meetings also provide students with information if they are on the right track. If they only find out at the end, then there is no time to make changes anymore.” *(Teacher interview “System Dynamics and Control” course)*

Furthermore, the progress meetings preceded the deadlines of the intermediate and final reports. The Process Dynamics & Control students reported to use the progress meetings to get feedback on their preliminary reports, both concerning the conducted experiments and the content of the reports. Since both reports were graded and contributed to passing the course, they activated the students to conduct experiments with the remote lab and report their results in a structured way. This aligns with the teacher’s intention as the progress reports served the goal of learning how to author a technical report.

The remote lab was introduced with a manual and a demonstration in the first week of the Process Dynamics & Control course. However, the students did not consider this very effective to engage them with the remote lab. They preferred a more active introduction in the form of a tutorial in which they could first-hand experience working with the remote lab.

“I would have preferred an active tutorial to practice with the remote lab. That would have saved us a lot of time spend on figuring it out by ourselves. Now it was a lot of trial-and-error. In the first week of the course there was a demonstration of the remote lab, but that went way too fast. […] I think I could learn more if I could actively practice with the lab compared to following a demonstration performed by someone else.” *(Student interview “System Dynamics and Control” course 2021)*

Furthermore, students indicated how getting familiar with the remote lab under guidance of a teacher could have supported their engagement. This was confirmed by the teacher’s observations that students were looking for directions and did not take initiative. The teacher acknowledged a need to change how the remote lab was introduced to students. She plans to replace the live demonstration with a video instruction on how to use the remote lab. The idea is that students can also use the video as a reference since the written manual designed for this purpose, was only used to a limited extent.

### Relationships

Students conducted the learning activities in teams of four or five students, which was reported to promote students’ engagement. The students from the Process Dynamics & Control course valued working in a team on the lab assignment, because it provided opportunities for discussion on the course content, remote lab experiments, results, and reports. According to the students, teamwork created opportunities for students to learn from their peers. However, the students also reported that they did not fully seize these opportunities for peer learning as they were inclined to distribute tasks and conduct these individually. Also, the distribution of tasks followed from students’ earlier experience with certain tasks. This means that students often opted for tasks that were close to their abilities and background, rather than maximizing opportunities to learn.

“Within our group we did not really have an official distribution of tasks, but if people have experience with something, for example with the use of Matlab, they take up these tasks because they can do them more easily and faster. In our group we often chose to assign a task to the person that could do it fast and well.” *(Student interview “System Dynamics and Control” course 2021)*

Furthermore, students most often worked on the remote lab assignments individually from home, especially in the Process Dynamics & Control course in the Spring of 2021 during Covid-19 regulations. According to the students, this minimized social relationships within their team. Team meetings were focused on substantive issues of the lab assignment and division of tasks, with considerably less (informal) social interaction compared to when they would work together in a physical lab. The students perceived this as a downside of working with a remote lab, because they valued social interaction and consequent relationships with peers.

“The group work was less personal. Meetings would quickly focus on what should be done. […] In the respect collaboration is more efficient, but you really miss the social interaction.” *(Student interview “System Dynamics and Control” course 2021)*

## Discussion and conclusion

The aim of this study was to identify how a remote lab assignment based on an active learning pedagogy in higher engineering education supports student engagement, with the overarching aim to promote students’ transfer skills from theory to practice. The results of this study show that an active learning pedagogy can support student engagement. Our results suggest the need for a structured arrangement of open-ended lab assignment, lab preparation, teamwork supporting peer learning and discussion, progress meetings focused on feedback and formative assessment, and reports focused on reflection.

Students reported a preference for the remote lab over the physical lab. However, evaluations also showed the need for support for amongst others, the process and the software use. Engagement can thus be strengthened by teacher support for students before and during the experiments, clear signposting for students about the experiment and lab set-up, and pre-structuring of lab activities. A clear process definition and active learning pedagogy, including amongst others a preparation phase, supported knowledge acquisition and enabled deep learning during and after experiments.

Because of the required investment in time and materials, it seems relevant to question what actual need a remote lab based on an active learning pedagogy fulfils. Opportunities for a remote lab include flexibility in scheduling experiments outside office hours. This allows students to plan and organize their learning activities and creates more opportunities to conduct (more) experiments (i.e., 24/7 instead of limited to office hours). However, this asks from students increased self-regulation and teamwork. On the other hand, physical laboratories require extensive preparation by students, and the risk of running out of time when something fails. Remote labs support breaks in the process to find required knowledge, and later continue the experiment. This freedom in turn allows for more open assignments, which aligns with open and flexible educational concepts, such as challenge-based learning (Gallagher & Savage, 2020).

### Physical space and technology

Implications of our case study for the development of remote labs regarding the physical space and technology, include the need for flexibility in scheduling of experiments. The 2020 course, which ran before the first Covid-19 lockdown, showed an intensive use of automated experiments in the evening and during the night. The 2021 run, during Covid-19 regulations, showed how students mostly ran live experiments, mainly during daytime. This could be caused by students being in lockdown at home and having more time available, for instance due to lack of travel times, lack of social activities, and shorter online lectures - typically 90 min - despite scheduling of the full physical lecture duration − 180 min.

The user friendly and intuitive interface increased student engagement with the remote lab. This aligns with earlier research showing that positive perceptions of a remote lab affect students’ perception and overall satisfaction, although not necessarily their learning outcomes (Viegas et al., 2018). Still, the availability of a live stream video camera also appears important for engagement and perceived interaction.

### Activities

Our results suggest how open-ended lab assignments create a learning urgency, which confirms existing engineering education research (Hernández-de-Menéndez et al., 2019). Yet, open-endedness implies flexibility, and possible procrastination for scheduling and conducting experiments. Students appear to be looking for directions, and even extrinsic triggers and rewards in the form of clear deadlines to avoid this procrastination. This, however, is at odds with the principle of self-regulated learning (Nascimento et al., 2019), but could also be the reason other studies find a lack of enthusiasm for active learning among engineering students (Shehkar et al., 2019; Weimer 2013)).

One central characteristic of the active learning approach in our case study was the role of progress meetings. They served as a deadline for students to actually get engaged, but also stimulated formative feedback by the teacher and discussion and reflection among the students concerning conducted experiments. This confirms existing research emphasising the role of formative assessment in engineering education (Gomez Puente, Van Eijck, & Jochems, 2013; Hassan 2011).

### Results

Both implicit learning goals, such as learning how an experiment set-up cannot be controlled manually, and explicit learning goals, such as learning how to build a controller and draft a technical report, are supported by the active learning pedagogy accompanying the remote lab. However, to ensure that students individually reach these learning goals, the remote lab requires a different physical experiment set-up every few years, because of the risk of students copying lab reports from previous years.

### Engagement

Implications for educational practice from the interplay between learning environment, learning activities, and relationships start with the way a remote lab is introduced to students. This introduction directly influences their engagement and resulting learning outcomes (Viegas et al., 2018). Our results suggest an interactive way of introducing the remote lab, rather than a demonstration or written manual. It can be considered as the first clear signposting for students (see also Van den Beemt et al., 2020).

Teamwork can be considered another aspect of this interplay resulting in engagement. When students were working from home, for example during Covid-19 regulations, they tended to perceive teamwork in a highly functional manner. Team meetings were focused on substantive issues of the lab assignment and division of tasks, with considerably less (informal) social interaction compared to when they would work together in a physical lab. Although the students perceived this as a downside of working with a remote lab, they still valued teamwork for the assignment, because it enabled discussion and peer learning. Paradoxically, our results also suggest that students had a purely functional approach to teamwork. Students applied a division of tasks and therein opted for tasks closest to their abilities and background, rather than maximizing opportunities to collaborate and learn together. Given the positive response to proposed deadlines, should teachers not provide another extrinsic trigger by pre-defining the division of tasks (see also Apul & Philpott 2011; Hamade & Ghaddar, 2011)?

Limitations to this evaluative case-study consist of a small number of courses in which the remote lab was applied, with, because of their specialised nature, only limited student enrollment. Interviews were held with only a small group of students, putting pressure on data saturation and generalizability of results (see also Guest et al., 2006). This implies that the results should be perceived as resonating with behaviour of other students instead of being generalised to the population at large. Hence, a follow-up study with more students might contribute to a better understanding of how an active learning pedagogy increases student engagement. Furthermore, the students were interviewed during the Covid-19 lockdown in Spring 2021, which might have affected for instance their view on teamwork and interpersonal communication.

Future research should address these issues, with multiple remote lab set-ups and a larger respondent group. This research could include a more in-depth analysis of the use of the remote lab, and reasons for why experiments might fail. Yet, this case study contributes to current developments, for instance in Challenge-based learning, which advocates open-ended assignments and flexible approaches as stimulating student engagement.
